# Evaluation of an Educational Health Website on Infections and Antibiotics in England: Mixed Methods, User-Centered Approach

**DOI:** 10.2196/14504

**Published:** 2020-04-06

**Authors:** Rosalie Allison, Catherine Hayes, Vicki Young, Cliodna A M McNulty

**Affiliations:** 1 Public Health England Gloucester United Kingdom

**Keywords:** user experience, usability, quality, online, science, health

## Abstract

**Background:**

e-Bug, an educational health website for teachers and students, aims to help control antibiotic resistance by educating young people about microbes, hygiene, and antibiotic resistance, reducing the incidence of infection and, therefore, the need for antibiotics. The teachers’ section of the e-Bug website has not been evaluated since it was launched in 2009, and worldwide page views have been steadily decreasing since 2013.

**Objective:**

This study aimed to apply GoodWeb, a comprehensive framework utilizing methodologies and attributes that are relevant to the digital era, to evaluate and suggest improvements to the e-Bug website.

**Methods:**

Electronic questionnaires and face-to-face completion of task scenarios were used to assess content, ease of use, interactivity, technical adequacy, appearance, effectiveness, efficiency, and learnability of the teachers’ section of the e-Bug website.

**Results:**

A total of 106 teachers evaluated the e-Bug website; 97.1% (103/106) of them reported that they would use e-Bug, and 98.1% (104/106) of them reported that they would recommend it to others. Participants thought that there was a niche for e-Bug because of the way the resources fit into the national curriculum. Suggestions for improvements included changing the menu indication by highlighting the current page or deactivating links, improving home page indication, and providing a preview of resources when hovering the mouse over hyperlinks. Additional features requested by users included a search function and access to training opportunities.

**Conclusions:**

This paper reports that the GoodWeb framework was successfully applied to evaluate the e-Bug website, and therefore, it could be used to guide future website evaluations in other fields. Results from this study will be used to appraise the current quality and inform any future changes, modifications, and additions to e-Bug.

## Introduction

### Background

According to Song and Zinkhan [1], an excellent website attracts more web users and encourages revisits if the user’s interests are carefully considered and incorporated in the design and presentation. User-centered design means that the websites can both fulfill the goals and desires of its users [2] and influence their perception of the organization and overall quality of resources [3].

There is no universally accepted method or technique for website evaluation; various assessment techniques have been employed to evaluate websites [4]. Both qualitative and quantitative measures are appropriate to evaluate website user experience. Questionnaires are the most widely used method for evaluating websites [5-11]. These can be administered remotely or in person [12] and used stand-alone or in combination with other methodology, such as observed browsing and interviews following completion of task scenarios [13,14]. Allison et al [7] incorporated methods and attributes from 69 studies to suggest a simple but comprehensive guide to evaluating websites, coined GoodWeb, which follows 4 basic steps:

- *Step 1*: What are the important website attributes that affect a user’s experience of the chosen website? For example, appearance, content, interactivity, ease of use, and technical adequacy
- *Step 2*: What is the best way to evaluate these attributes? For example, questionnaire and observed completion of task scenarios
- *Step 3*: Who should evaluate the website? For example, users
- *Step 4*: What setting should be used? For example, face-to-face/controlled and remote

### The Website

e-Bug [15] is an ongoing international project, operated by Public Health England (PHE), that creates health education resources for teachers and students, covering the subjects of microbes, hygiene, and antibiotic use and resistance [16]. All activities and plans have been designed to complement the national curriculum, particularly Biology and Personal, Social, Health, and Economic [15]. The National Institute for Health and Care Excellence (NICE) recommended that schools could use e-Bug when teaching about antibiotics and infections [17], and e-Bug is a case study in the UK 5-year Antimicrobial Resistance Strategy 2019-2024 [18]. A key component of the e-Bug project is the e-Bug website, established in September 2009 [15]. The e-Bug website comprises 2 microsites: an educator microsite that includes free teaching resources, such as lesson plans and student worksheets [16], and a student microsite that hosts interactive activities, games, and animations, which are developed by graphic designers, researchers, and microbiologists. The e-Bug resources, in particular, the digital media, including the games, have been well evaluated [19,20]. Worldwide page views of the teachers’ section have been steadily decreasing since 2013 (282,284 views in 2013-2014, 248,260 views in 2014-2015, 208,540 views in 2015-2016, and 197,740 views in 2016-2017) [21]. The fact that e-Bug is recognized by both NICE [17] and the Department of Health [18] as a recommended tool for teachers highlights the importance of this evaluation to continually strive to improve this part of the website to aid implementation.

In 2015, hard-copy e-Bug resources were sent to all schools in England [22], and the e-Bug team regularly promoted the resources at popular teacher conferences, such as Big Bang and Association for Science Education (ASE) conferences. The circulation of hard-copy resources may be one explanation for the decrease in page views, but a formal evaluation of the teachers’ website may provide other explanations and inform improvements.

### Aims

This study aimed to apply GoodWeb, a comprehensive guide, to evaluate an educational health website, using recognized methods to evaluate the most crucial website attributes. The results of the evaluation will be used to appraise the current website quality, inform any future modifications or additions to the website, and the possibility of a full-scale evaluation of the whole site. In addition, the evaluation will be used to advise whether GoodWeb is an effective and appropriate website evaluation guide.

## Methods

### Study Design

Following GoodWeb, this evaluation was based on the most appropriate existing methodologies and techniques to evaluate websites, determined by a robust review of the current website evaluation literature [7]. The evaluation included a 2-part questionnaire (Multimedia Appendix 1). Part A collected baseline data, whereby users were asked users to rank the importance of different website attributes under the categories appearance, content, interactivity, ease of use, and technical adequacy and then score the e-Bug website against these attributes. Part B of the questionnaire allowed users to rate e-Bug’s performance against the attributes ranked in part A. In addition, a subset of users completed website task scenarios to provide more in-depth feedback on the use of the e-Bug website. These data were used to inform prioritization for changes to the e-Bug website.

To assess feasibility and gain suggestions for improvements to the study design, the questionnaire was piloted with 36 users and the task scenarios with 14 users at a 2-day conference. Changes and decisions made based on the pilot and feasibility study include providing the questionnaire in an electronic format rather than a paper-based format, finalizing which website attributes to evaluate, recording the task completion as there was too little time to make comprehensive notes and record mouse clicks at the same time, and providing tick box options to minimize free-text comment boxes.

### Recruitment

**Figure 1:**
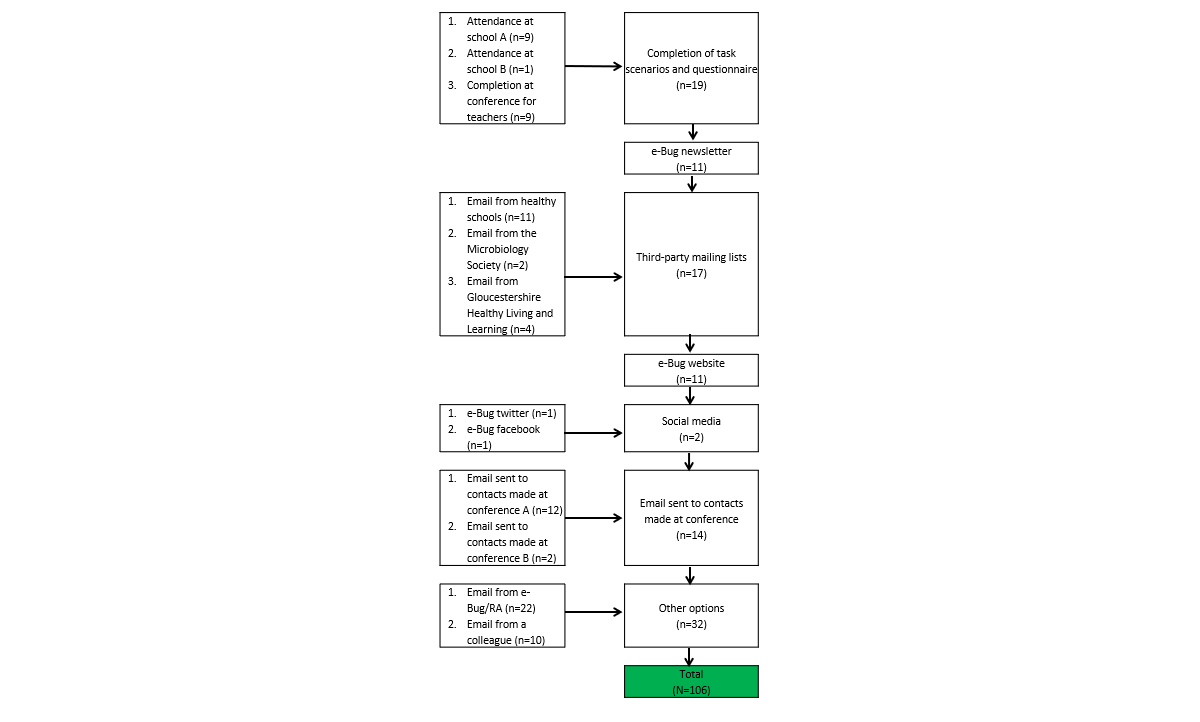
Recruitment flowchart. RA: Rosalie Allison.

To increase heterogeneity of participants, for example, to incorporate a range of geographical locations in England, different experiences of e-Bug and to include primary and secondary school teachers, participants were recruited by email from e-Bug contact lists and face-to-face meetings at educational conferences (see Figure 1 for recruitment flowchart).

### Questionnaire Participant Recruitment

Participants were sent a link to the electronic questionnaire, hosted on SelectSurvey, via teacher mailing lists from e-Bug, healthy schools’ leads, At-Bristol Science Centre, Microbiology Society, and Gloucestershire Healthy Living and Learning (GHLL); the e-Bug website’s teachers’ home page; e-Bug’s Twitter and Facebook pages; and teacher contacts that visited the e-Bug advertorial stand at conferences.

### Completion of Task Scenarios Recruitment

Teachers visiting the e-Bug stand at 2 conferences (Big Bang 2016 and ASE 2017) were asked whether they would like to participate in an e-Bug website evaluation. Interested teachers were provided with an information sheet, given the opportunity to ask questions, and ensured that they were comfortable with the environment and surroundings, before informed written consent was obtained. Some teachers participated at the conference; others were visited in their own school.

As an incentive to participate in the website evaluation, all participants were offered a £5 (US $6.50) high street gift voucher and a professional certificate for Continuing Professional Development. Participants that completed the additional component of task scenario completion were offered e-Bug resources. Questionnaire participants were entered into a draw to win a set of giant microbes.

### Data Collection

#### Questionnaire Part A: Baseline Questionnaire

Before assessing the e-Bug website, all 106 participants completed an electronic questionnaire (Part A - Multimedia Appendix 1), hosted on SelectSurvey, which asked general questions, including the following:

- Demographic information, such as role, for example, teacher and age groups they teach
- Which teaching resources participants currently use and what devices they use to access resources
- Ranking the 5 main website attributes (appearance, content, interactivity, ease of use, and technical adequacy) in order of importance to themselves as educators. Then within each main attribute, ranking the importance of the subcategories, which ranged from between 2 and 6 subcategories within each main attribute.

### Familiarity With e-Bug

All 106 participants were provided with the website link and asked to familiarize or refamiliarize themselves with the teachers’ section of the e-Bug website. After 5 min of exploring the e-Bug website, participants completing the questionnaire remotely (n=87) moved on to questionnaire part B.

#### Completion of Task Scenarios

The 19 participants that were face-to-face with the researchers (RA and CH) at the school or conference were asked to complete task scenarios. Participants were provided with scenarios, typical of users of the e-Bug website, and asked to think aloud [23] as they attempted to complete the task. The screen recording function on Skype for Business was used to record participants’ website navigation and capture audio output. Multimedia Appendix 2 shows the task scenarios the participants were asked to complete. Researchers (RA and CH) made observational notes and probed about the ease of completion and ideas for improvement after each task scenario. For the task completion, some teachers participated at a conference; others were visited in their own school.

#### Questionnaire Part B

All 106 participants completed an electronic questionnaire (Part B - Multimedia Appendix 1), hosted on SelectSurvey. This electronic questionnaire included the following:

- Rating (5-point Likert scale) the e-Bug website against all website attributes previously ranked by them in part A: baseline questionnaire by indicating how strongly they agreed or disagreed with statements about the website, for example, “” (strongly disagree/disagree/neither agree or disagree/agree/strongly agree).
- Overall satisfaction with the website, including ideas for improvement
- Loyalty, measured by their enthusiasm to use e-Bug again and/or inclination to recommend to a colleague/friend
- Importance to participants of suggestions of some additional content, for example, search function; ability to like, share on social media, or email specific e-Bug resources to a friend; and development of an app for users.

### Analysis

#### Task Scenarios

The task scenarios were used to analyze the following [24]:

- Effectiveness (whether it is possible to complete the realistic tasks for end users, which is measured by the percentage of tasks completed)
- Efficiency (whether end users are able to locate the resources using the quickest and most direct route through the website, which is measured by the number of “additional” clicks to locate resources)
- Learnability (whether the structure of e-Bug’s website is easy to remember for future use, which is measured by the change in efficiency in the repeated task).

#### Questionnaire

**Figure 2:**
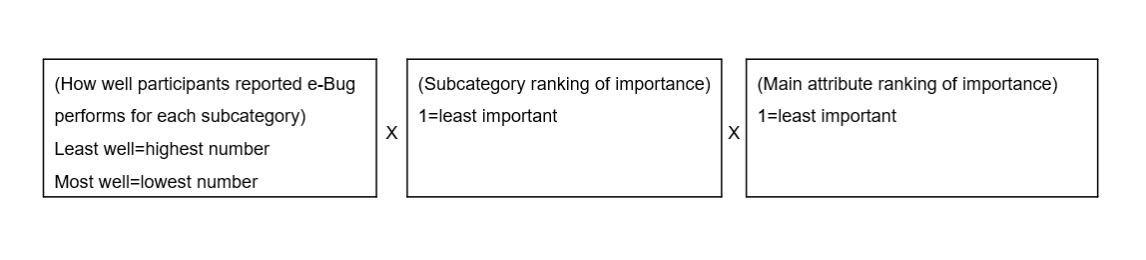
Calculation to combine importance of an attribute and performance.

Participants’ responses to ranking the importance of website attributes and rating the e-Bug website against these attributes were combined and averaged to provide a prioritization order for implementing change [25]. This was done as follows:

1. Post data collection, the ranking importance given to the 5 main attributes (appearance, content, interactivity, ease of use, and technical adequacy) and their subcategories was reversed so that the least important categories scored only one. This meant that, when calculated, the largest number indicated the highest priority for change.
2. For each subcategory, multiply the rating by the subcategory ranking by the category ranking (Figure 2).
3. Calculate the mean average for the respondents.
4. Divide by the number of subcategories within the main attribute to account for the differing number of subcategories in each main attribute group.
5. Order in descending order—highest number is the highest priority for change.

A random number generator on Excel was used to calculate a value for compatibility with other devices, guidance, sense of community, modern features, and limited use of special plug-ins, as it was not possible for participants to rate these attributes because of the fact that the features did not currently exist, for example, guidance, sense of community, and modern features, or because of the fact that, in a controlled environment, participants were not asked to browse the website on different devices. It was necessary to assign a value to these attributes so as to be included in the overall order for prioritization of change.

Descriptive statistics were used to analyze the remaining questionnaire data, and the free-text answers were collated and qualitatively analyzed, in combination with the transcribed audio output from completion of the task scenarios, using NVivo 10, to provide the main themes. These were discussed and agreed upon by the project team.

### Ethical Approval

This evaluation was approved by the PHE Research Ethics and Governance Group (REGG). After review, the Research Governance Coordinator for PHE confirmed that no ethical approvals were needed.

## Results

### Overview

**Figure 3:**
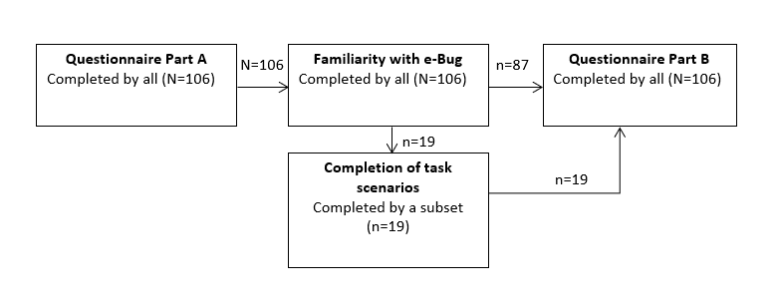
Visual summary of data collection process.

A total of 19 participants completed the task scenarios and the questionnaire. Moreover, 87 participants completed the questionnaire only, resulting in a total of 106 heterogeneous participants evaluating the e-Bug website (see Figure 3 for the visual summary of the data collection process and Multimedia Appendix 3 for an overview of participants and the devices used to access teaching resources).

A total of 97.1% (103/106) of the participants would use the e-Bug website in the future, and 98.1% (104/106) of the participants would recommend it to a colleague or friend. Moreover, 65.0% (67/103) of the participants that indicated that they would use e-Bug again were first-time users of the website. A yes or no option button within the questionnaire part B was appropriate for the quantitative element of this attribute, with an additional open comment box for participants to provide reasons for their answers.

Participants’ commented as follows:

Great website which has been highly effective in supporting the new science GCSE.

Teacher, Secondary

Definitely a niche in the market for it...useful to have a site that does all and that makes it applicable and user friendly for children.

Assistant Head Teacher, Primary

Multimedia Appendix 4 shows users’ comments about the e-Bug website.

### Completion of Task Scenarios

Table 1 shows the effectiveness (whether it is possible to complete the realistic tasks for end users, which is measured by the percentage of tasks completed) and Table 2 shows the efficiency (whether end users are able to locate the resources using the quickest and most direct route through the website, which is measured by the number of “additional” clicks to locate resources) of the e-Bug website. Moreover, 100% (19/19) of the participants were able to locate the full pack of resources (task 1) and the link to the national curriculum (task 5). Participants found it most difficult to return to the e-Bug home page when they had navigated away from it, as only 59% (10/17) of the participants were able to locate this, suggesting that this is an obvious area for improvement of the website.

**Table 1:** Effectiveness of the e-Bug website. Tasks are arranged from most effective at the top to least effective at the bottom (N=17).

Task number	Description of task	Effectiveness of participants that were able to complete the task, n (%)
Task 1	Full pack of resources	17 (100)
Task 5	National curriculum	17 (100)
Task 7	Task 1 again—full pack of resources	16 (94)
Task 3	Vaccinations timeline	14 (82)
Task 4	Antibiotic worksheet	14 (82)
Task 2	Hand hygiene complete pack	13 (77)
Task 6	e-Bug home page	10 (59)

**Table 2:** Efficiency of the e-Bug website. Tasks are arranged from most efficient at the top to least efficient at the bottom.

Task number	Description of task	Efficiency (average number of additional clicks participants needed to complete the task)
Task 3	Vaccinations timeline	0.1
Task 7	Task 1 again—full pack of resources	0.2
Task 5	National curriculum	0.2
Task 2	Hand hygiene complete pack	0.3
Task 4	Antibiotic worksheet	0.7
Task 1	Full pack of resources	0.8
Task 6	e-Bug home page	1.4

Most participants were able to complete scenarios typical of an e-Bug user. The following feedback was included:

Really easy to navigate around. I’m sure even a technophobe would be able to do it.

Teacher, Secondary

Possibly the fact I that had looked at the website previously, even just for a couple of moments, it is quite intuitive because each page is laid out in the same way. I know that I can get the whole pack and then individual resources: teacher’s sheets, pupil’s sheets. Funny, because I put consistency as quite low [referring to part A: baseline questionnaire], but seeing how consistent e-Bug is proves that this is actually quite important to me.

Teacher, Primary

Very useful resources and tools. Nice and teacher friendly. Doesn’t require a massive amount of time browsing to find the information you need.

Teacher, Secondary

Participants were able to locate the vaccination timeline (task 3) most efficiently, with minimal unnecessary clicks around the website. Conversely, participants found locating the e-Bug home page most difficult (task 6). Those that were able to locate it (only 10/17, 59%) took, on average, an extra 1.4 clicks compared with the most efficient route, with 0-4 additional clicks needed to locate the e-Bug home page.

Feedback and suggestions for improvement included the following:

Would never think to click on the logo. Have a pop-up description when hover over with mouse. Call teacher’s home page, teacher’s hub.

Teacher, Secondary

Everything else is words so wasn’t looking for a symbol.

Teacher, Primary

Not consistent with “young adult page.” When scrolling over, it doesn’t come up with “hand image”- noticed when surfing the websites. And this does not take you back to the e-Bug home page, so very confusing.

Teacher, Secondary

Expected home link to take to the e-Bug home.

Teacher, Secondary

In comparison with other task scenarios, participants also struggled to find the most efficient route to the full pack of resources (task 1—required an additional 0.8 clicks on average, range of 0-3 additional clicks) and the antibiotic worksheet (task 4—required an additional 0.7 clicks on average; range of 0-8 additional clicks). When finding the full pack of resources, participants were unaware that two separate links took them to the same page, and therefore, they often clicked both options. A suggestion to alleviate this included the following:

It would be useful that, when on a page, the link in the menu goes a different colour. Or when on a certain page, deactivate the link in the menu, so that you know you’ve actually gone to that page.

Teacher, Secondary

To increase the efficiency of finding specific worksheets, such as the student worksheet on antibiotics (task 4), suggestions included:

Perhaps when hover mouse over link, a dialogue box with info on each link could come up.

Teacher, Secondary

Would be useful to have a print-screen or image of what the worksheet looked like. Or rollover image...interactive feature.

Teacher, Secondary

Owing to the small sample size and the high efficiency and effectiveness of task scenario completion first time around, it was not possible to statistically analyze learnability (whether the structure of e-Bug’s website is easy to remember for future use), which is measured by change in effectiveness and efficiency in repeated tasks (comparing tasks 1 and 7).

However, qualitative feedback included:

Having done one, it then became clear what to do with the other ones. Easy to learn.

Teacher, Secondary

A LOT easier than previous time. Would remember in a month’s time. Practice made navigation easier.

Teacher, Secondary

### Ranking Importance of Website Attributes

**Figure 4:**
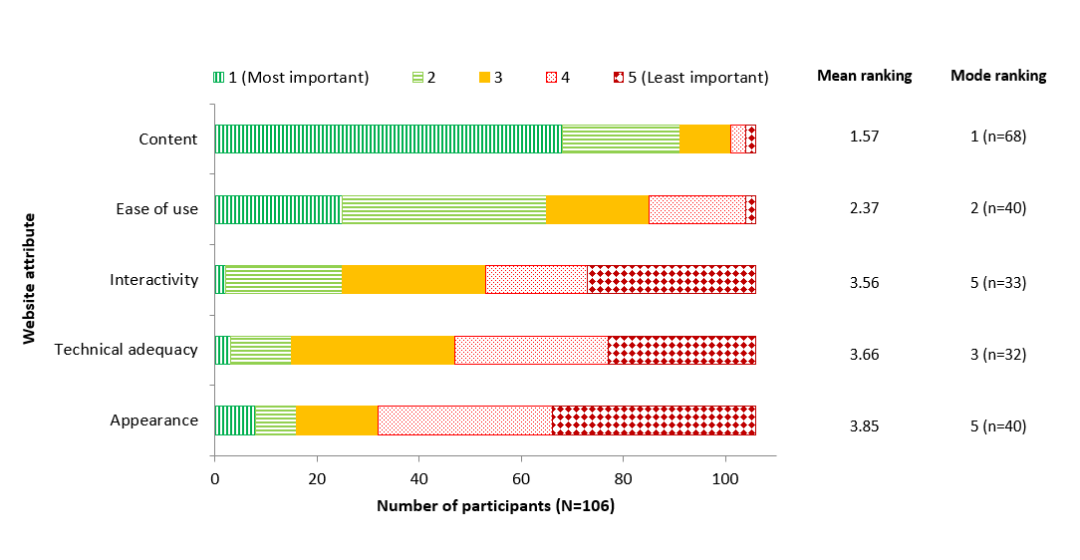
Importance of the five main website attributes to teachers.

On average, participants ranked the content (68/106, 64.1% participants) of the website as the most important website attribute to themselves as educators and appearance (40/106, 37.7% participants) as the least important attribute (Figure 4). For ranking of all subcategories, see Multimedia Appendix 5.

Reasons given for ranking content as the most important attribute included:

It doesn’t matter how flashy the website, if the content is wrong then it’s no good.

Teaching Assistant, Secondary

If the content is not correct, relevant, or detailed enough, then it is useless to me despite how nice it may look, or how easy it is to use.

Teacher, Secondary

Reasons given for ranking appearance as the least important attribute included:

Whilst the appearance is important to attract people, I am more interested in appropriate, easily accessed information.

Teacher, Secondary

I don’t really care what it looks like, so long as I can adapt resources.

Teacher, Secondary

### Rating e-Bug Against the Website Attributes Previously Ranked

**Figure 5:**
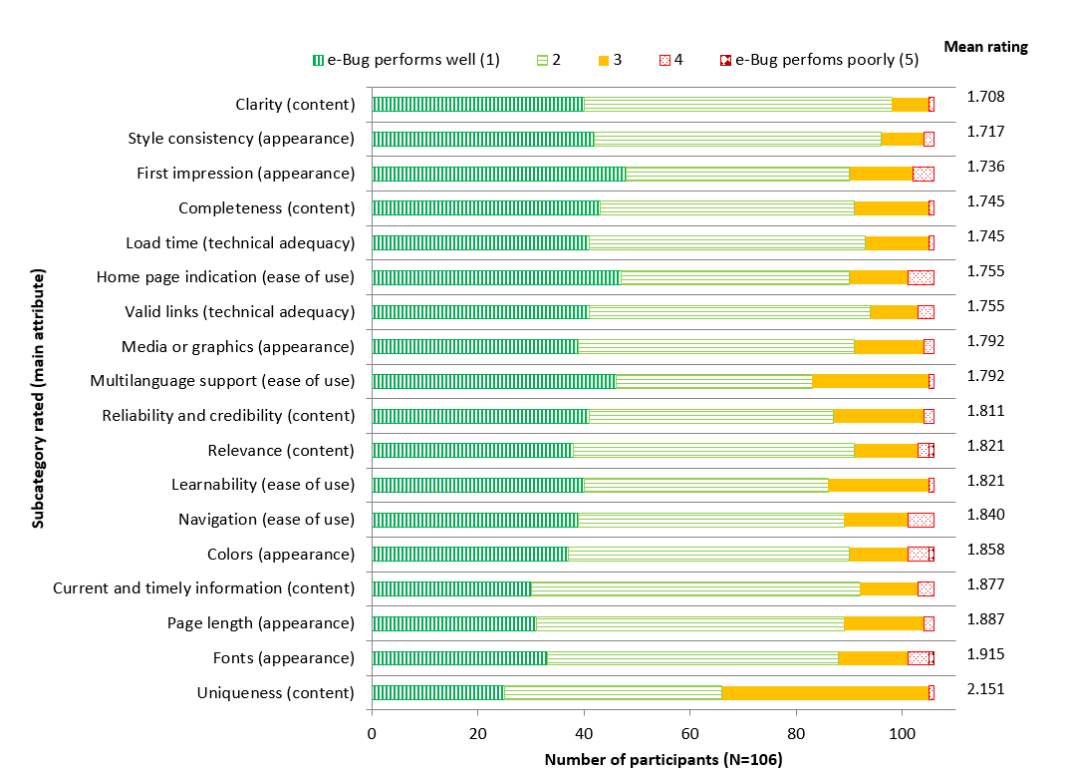
Users’ rating of the teachers’ section of the e-Bug website in regards to different website attributes.

Figure 5 shows that overall, e-Bug performs very well, as all averages of the 18 attributes evaluated ranged between 1.708 and 2.151, with 1 being the best and 5 being the worst on the Likert scale. Clarity of content, style consistency, and first impression were rated as e-Bug’s best qualities. Attributes that e-Bug was rated lowest against included uniqueness of content, fonts, and page length, but they were still rated positively on the Likert scale (<3).

### Prioritization for Change

**Figure 6:**
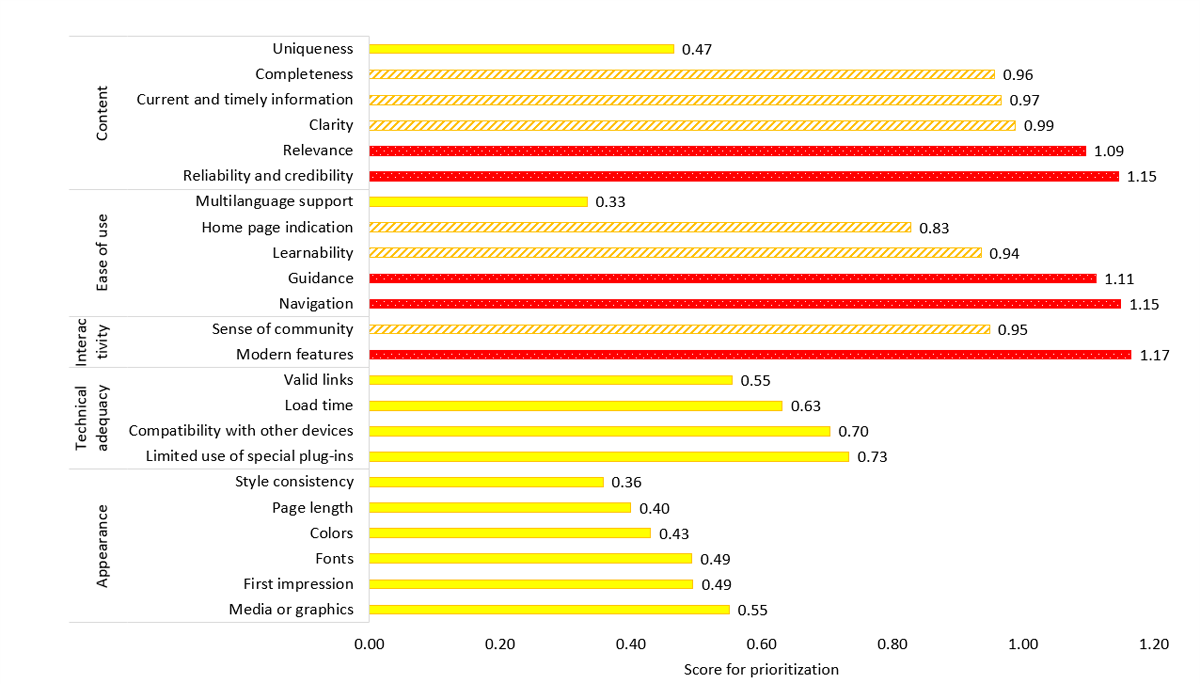
Prioritization score combining users' ranking of attributes' importance and rating of e-Bug's performance with red dots representing the highest priority for change, orange diagonal stripes lower priority, yellow block lowest priority.

Figure 6 shows the priority score for implementing changes, based on users’ perception of importance of attributes combined with how well e-Bug performed against these attributes. The highest priorities for change, highlighted in red in Figure 6, included modern features: the educational website reflects the most current trend or trends, eg, Twitter feeds visible and blog posts; navigation: navigating the educational website is intuitive, and it is easy to find the desired information; reliability and credibility: the educational website provides information that is trustworthy; guidance: the educational website provides help for users in recovering from common errors or assists them in the completion of tasks, eg, frequently asked questions, help option, and search tool; and relevance of content: the educational website offers content that is relevant to educators. The attribute of least priority is multilanguage support (the educational website supports its users’ language preferences), which corresponds with the fact that e-Bug is available in 23 different languages.

Suggestions to improve reliability and credibility of e-Bug’s content include:

Cite where the information given has come from. Clearly show the year the information was updated.

Science Technician, Secondary

See Multimedia Appendix 6 for participants’ suggestions for improvement, using the prioritization order for change as a framework.

### Prioritization for Additional Features

**Figure 7:**
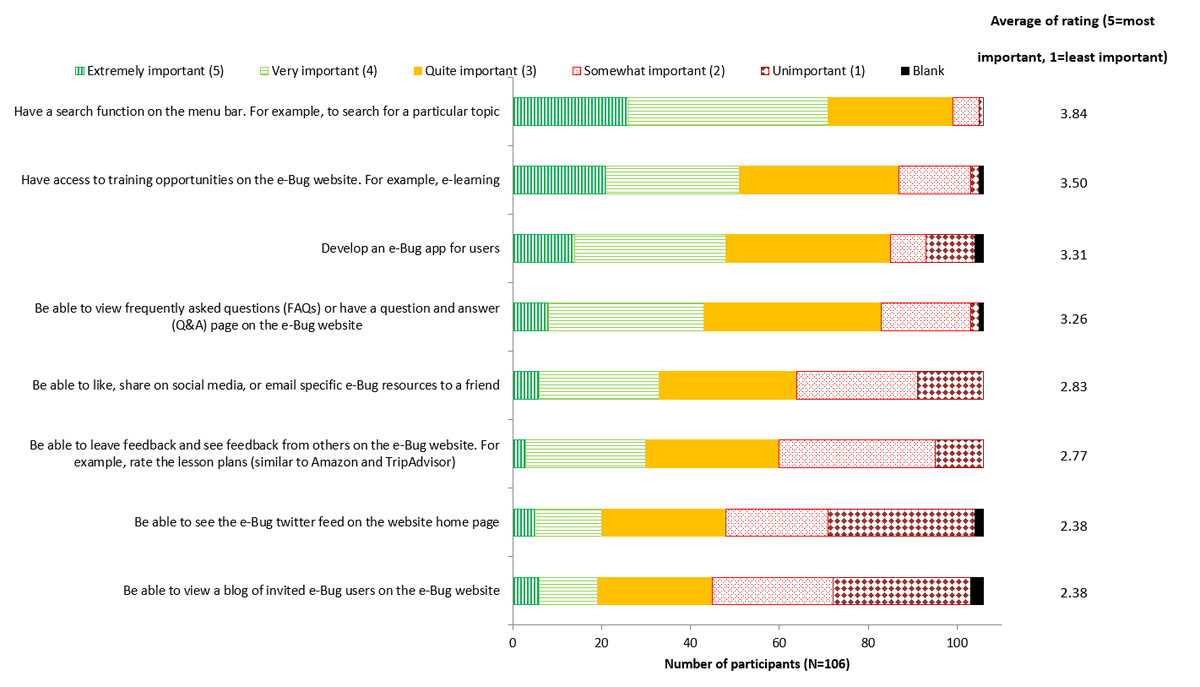
Importance of additional features to the teacher’s section of the e-Bug website.

Figure 7 shows the priority order for additional features to the teachers’ section of the e-Bug website. Of features that e-Bug does not currently have, the highest priorities, rated by the users, include a search function (rated as “extremely important” or “very important” by 66.9% [71/106] of participants) and access to training opportunities (rated as “extremely important” or “very important” by 48.1% [51/106] of participants).

## Discussion

### Principal Findings

Overall, teachers liked the e-Bug website; 97.1% (103/106) of them would use it themselves, and 98.1% (104/106) of them would recommend e-Bug to others. Feedback showed that there are a lot of websites with science resources, but there was a niche for e-Bug because of the bulk of resources that fit into the national curriculum.

This study found that, for educational websites, users value the content of the website over other attributes, such as appearance (Figure 4). Teachers reported that they often take the content from educational health websites and locally modify it to fit their purpose or ability of the audience. Website designers of health care websites should be mindful of this when designing future platforms.

Findings from the completion of typical task scenarios with users of the e-Bug website were that it is generally easy to navigate and find resources, even with little previous experience on the website. Participants were able to locate the vaccination timeline efficiently, with minimal unnecessary clicks around the website, suggesting optimal information architecture [2] of this page. However, as participants were less able to return to the home page, they may have missed the whole student’s section of the website, which includes games, revision guides, and quizzes for students to play, learn from, and complete as well as resources for communities and training for educators.

In terms of other improvements to e-Bug, features such as having a search function; developing an e-Bug app; and being able to like, share on social media, or email specific e-Bug resources to a friend could improve the “modern features” attribute, voted as the highest priority for change, and the “sense of community,” ranked in the ninth position for priority for implementation. e-Bug was originally developed in 2009, and this study has clarified that the technology is out of date as there has been a shift in the digital market [26]. Further research is needed in this area to see how new media can better support teachers and educational providers.

### Strengths and Limitations of GoodWeb

After piloting GoodWeb to evaluate the e-Bug website, it can be said that a strength of the framework is the easy step-by-step guide, which is adaptable and flexible to the website and objective of the evaluation.

Previous evaluations of health websites focus specifically on the quality of the content [27]. This study takes a holistic approach assessing the quality of the website as a whole, facilitated by the step-by-step guide of GoodWeb [7], which highlighted areas for improvement that would not otherwise have been identified.

A possible limitation is that the study design did not allow for “learnability” to be measured, because of the small sample size and the high efficiency and effectiveness of task scenario completion first time around. It would be advised that a larger sample size is required for this attribute, or conversely, more complex task scenarios, although this was not appropriate for e-Bug as most of the typical tasks are relatively simple.

A major strength of this study is the methodology chosen of both ranking the importance of attributes and then rating how well e-Bug performed against these attributes, which meant that it was possible to calculate an overall prioritization order for change. In addition, using a range of end users to evaluate the website, including those who were familiar with e-Bug and first-time users as well as participants with varying levels of computer literacy, means that e-Bug can be tailored to the needs of all end users.

A possible limitation of the methodology is that only a subset of participants completed the task scenarios. This decision was made because of the time taken for task completion and the logistics of the researchers observing, in person. If the study were to be repeated, the researchers could use a combination of observing the task scenarios in person and remotely, for example, utilizing the “share desktop” and “record” functions of Skype for Business. In this study, screen capture with audio output was essential to capture the task scenario data, as it allowed the researcher to assess efficiency, effectiveness, and learnability at a later time, and pull out and compare key themes from the discussion between the user and researcher, without compromising the situation at the time with excessive note taking and counting of mouse clicks. This process provided such rich data from just a subset of participants, that it was not deemed essential for all participants to complete.

A possible limitation of this evaluation is the small sample size, in comparison with larger studies [12]. However, there is still uncertainty over how many participants are needed to assess usability [28-32]. With the addition of the in-depth observational data during and interviewing post completion of task scenarios, data collected were rich and valuable for appraising quality and suggesting modifications for implementation, and the data were not dissimilar to other studies [33-38].

A constraint of this methodology is that subcategories could not be compared with subcategories from other categories, that is, ranking of clarity of content could not be compared with ranking of compatibility with other devices as they are within different categories (content compared with technical adequacy). Feedback from the feasibility study suggested that participants were unlikely to rank the 23 attributes assessed using the questionnaire, in order of importance, and therefore, the chosen methodology reflects the advice of users to successfully attain a high completion rate. This highlights the importance of patient and public involvement [39,40] throughout the evaluation process.

Furthermore, a random number generator was used to rate the attributes for features that did not currently exist, which could have introduced inaccuracies. If the study were to be repeated, it is advised that an average of other ratings is used instead, to account for this.

### Recommendations

Areas that users ranked as most important and where e-Bug is currently not delivering as well as it could include modern features, navigation, reliability and credibility of content, guidance, and relevance of content.

The highest priority for additional features that e-Bug does not currently have include a search function and access to more training opportunities, which may improve the subcategory “modern features.” As a result, e-Bug has already reacted to these findings and now advertises e-learning modules and face-to-face e-Bug–approved educator training, through the e-Bug website. This has been successful as e-Bug has now trained over 100 educators (2016-2018), as advertising on the website and page views have started to increase (220,045 views in 2017-2018).

Users’ suggestions to make the website even easier to navigate include highlighting or deactivating the current page in the menu bar, improving home page indication, and a preview of resources when hovering the mouse over hyperlinks.

Suggestions to improve reliability and credibility of content were to cite where the information had come from and clearly show the year that the content had last been updated.

By implementing the suggested changes and continuing to promote e-Bug, it is hoped that the trend of reduced use of the teachers’ pages will be reversed and current and new users will be retained. It is recommended, therefore, that the e-Bug website is evaluated again, following the implementation of the suggested modifications.

Finally, GoodWeb, the comprehensive framework, was successfully applied to evaluate this educational health website and, therefore, could be used to guide future website evaluations in other fields.

## Appendix

Multimedia Appendix 1 e-Bug website evaluation questionnaire.

Multimedia Appendix 2 Task scenarios.

Multimedia Appendix 3 Overview of participants and devices used to access teaching resources.

Multimedia Appendix 4 User’s comments about the e-Bug website.

Multimedia Appendix 5 Ranking importance of sub-categories within categories to teachers.

Multimedia Appendix 6 Participants’ suggestions for improvement, using the prioritisation order for change as a framework.

## Abbreviations

NICE: National Institute for Health and Care Excellence
PHE: Public Health England

## References

[1] Song J, Zinkhan G (2003). Features of web site design, perceptions of web site quality, and patronage behavior.

[2] Mashable.

[3] Gonzalez ME, Quesada G, Davis J, Mora-Monge C (2015). Application of quality management tools in the evaluation of websites: The case of sports organizations. Qual Manage J.

[4] Tsai WH, Chou WC, Lai CW (2010). An effective evaluation model and improvement analysis for national park websites: A case study of Taiwan. Tour Manag.

[5] Barnes SJ, Vidgen RT (2006). Data triangulation and web quality metrics: A case study in e-government. Inf Manag.

[6] Thielsch MT, Blotenberg I, Jaron R (2014). User evaluation of websites: From first impression to recommendation. Interact Comput.

[7] Allison R, Hayes C, McNulty CA, Young V (2019). A comprehensive framework to evaluate websites: literature review and development of GoodWeb. JMIR Form Res.

[8] Hartmann J, Sutcliffe A, Angeli AD (2008). Towards a theory of user judgment of aesthetics and user interface quality. ACM Trans Comput-Hum Interact.

[9] Bargas-Avila JA, Hornbæk K (2011). Old Wine in New Bottles or Novel Challenges: A Critical Analysis of Empirical Studies of User Experience. Proceedings of the SIGCHI Conference on Human Factors in Computing Systems.

[10] Hassenzahl M, Tractinsky N (2006). User experience - a research agenda. Behav Inf Technol.

[11] Aranyi G, van Schaik P (2016). Testing a model of user-experience with news websites. J Assn Inf Sci Tec.

[12] Elling S, Lentz L, de Jong M, van den Bergh H (2012). Measuring the quality of governmental websites in a controlled versus an online setting with the ‘Website Evaluation Questionnaire’. Gov Inf Q.

[13] Chase D, Trapasso E, Tolliver R (2016). The perfect storm: examining user experience and conducting a usability test to investigate a disruptive academic library web site redevelopment. J Web Librarianship.

[14] Petrie H, Precious J (2010). Measuring User Experience of Websites: Think Aloud Protocols and an Emotion Word Prompt List. Proceedings of the 28th Annual CHI Conference on Human Factors in Computing Systems.

[15] e-Bug.

[16] McNulty CA, Lecky DM, Farrell D, Kostkova P, Adriaenssens N, Herotová TK, Holt J, Touboul P, Merakou K, Koncan R, Olczak-Pienkowska A, Avô AB, Campos J, e-Bug Working Group (2011). Overview of e-Bug: an antibiotic and hygiene educational resource for schools. J Antimicrob Chemother.

[17] Regis T, Stone J (2017). The National Institute for Health and Care Excellence.

[18] Department of Health and Social Care (2019). UK Government.

[19] Eley CV, Young VL, Hayes CV, Verlander NQ, McNulty CA (2019). Young people's knowledge of antibiotics and vaccinations and increasing this knowledge through gaming: mixed-methods study using e-Bug. JMIR Serious Games.

[20] Hale AR, Young VL, Grand A, McNulty CA (2017). Can gaming increase antibiotic awareness in children? a mixed-methods approach. JMIR Serious Games.

[21] Young VL, Rajapandian V, Eley CV, Hoekstra BA, Lecky DM, McNulty CA (2015). Monitoring web site usage of e-Bug: a hygiene and antibiotic awareness resource for children. JMIR Res Protoc.

[22] Eley CV, Young VL, Hoekstra BA, McNulty CA (2017). An evaluation of educators’ views on the e-Bug resources in England. Journal of Biological Education.

[23] Yen PY, Bakken S (2009). A comparison of usability evaluation methods: heuristic evaluation versus end-user think-aloud protocol - an example from a web-based communication tool for nurse scheduling. AMIA Annu Symp Proc.

[24] Arrue M, Fajardo I, Lopez JM, Vigo M (2007). Interdependence between technical web accessibility and usability: its influence on web quality models. Int J Web Eng Technol.

[25] Agarwal R, Venkatesh V (2002). Assessing a firm's Web presence: a heuristic evaluation procedure for the measurement of usability. Inf Syst Res.

[26] Budiu R, Nielsen J (2015). Semantic Scholar.

[27] Eysenbach G, Powell J, Kuss O, Sa E (2002). Empirical studies assessing the quality of health information for consumers on the world wide web: a systematic review. J Am Med Assoc.

[28] Faulkner L (2003). Beyond the five-user assumption: benefits of increased sample sizes in usability testing. Behav Res Methods Instrum Comput.

[29] Hwang W, Salvendy G (2010). Number of people required for usability evaluation: the 10±2 rule. Commun ACM.

[30] Macefield R (2009). How to specify the participant group size for usability studies: a practitioner's guide. J Usability Stud.

[31] Nielsen J (1994). Usability Engineering.

[32] Virzi RA (1992). Refining the test phase of usability evaluation: how many subjects is enough?. Hum Factors.

[33] Muhtaseb R, Lakiotaki K, Matsatsinis N (2012). Applying a multicriteria satisfaction analysis approach based on user preferences to rank usability attributes in E-tourism websites. J Theor Appl Electron Commer Res.

[34] Moreno JM, del Castillo JM, Porcel C, Herrera-Viedma E (2009). A quality evaluation methodology for health-related websites based on a 2-tuple fuzzy linguistic approach. Soft Comput.

[35] Leite P, Gonçalves J, Teixeira P, Rocha A (2014). Towards a model for the measurement of data quality in websites. New Rev Hypermedia Multimedia.

[36] Flavián C, Guinalíu M, Gurrea R (2006). The role played by perceived usability, satisfaction and consumer trust on website loyalty. Inf Manag.

[37] Emmert M, Adelhardt T, Sander U, Wambach V, Lindenthal J (2015). A cross-sectional study assessing the association between online ratings and structural and quality of care measures: results from two German physician rating websites. BMC Health Serv Res.

[38] Deng J, Zhang T, Li M (2011). The Empirical Analysis of Design Quality Measurement System Concerning Tour Electronic Commerce Website in China. Proceedings of the 2011 International Conference on E-Business and E-Government.

[39] Brett J, Staniszewska S, Mockford C, Herron-Marx S, Hughes J, Tysall C, Suleman R (2014). Mapping the impact of patient and public involvement on health and social care research: a systematic review. Health Expect.

[40] NIHR Involve.

