# Supplementary material for: Evaluation of an Educational Health Website on Infections and Antibiotics in England: Mixed Methods, User-Centered Approach
Source: JMIR Form Res. 2020 Apr 6;4(4):e14504. doi: 10.2196/14504 (PMC7171564; doi:10.2196/14504)
Supplement: Multimedia Appendix 1 [file formative_v4i4e14504_app1.docx]

e-Bug website evaluation

**What do users think of the teacher's site of the e-Bug website?**

**We are looking to evaluate our website to see what improvements are needed to make it the best website for our audience’s needs. We do hope you will therefore take just a few minutes to answer the following short questions.**

**PART A.**

**1. What is your role?** (Tick **one**)

🞏Teacher 🞏Teaching assistant 🞏School Nurse 🞏Other educator

If other, please specify...................................................................................................................................................

**2. What age groups do you cover?** (Tick all that apply)

🞏KS1 🞏KS2 🞏KS3 🞏KS4 🞏KS5

**3. Gender 4. Location of your school** (please enter the nearest town/city)

🞏Male 🞏Female 🞏Would rather not say .........................................................................................................

**5. Age**

🞏24 or below 🞏25-35 🞏36-45 🞏46-55 🞏 56 or over

## Promotion or availability of educational resources

**6. Where do you currently access teaching resources?** (Tick all that apply)

🞏Times Educational Supplement (TES) 🞏Guardian Teaching Network (GTN)

🞏Social media groups 🞏Yahoo groups

🞏Subscriptions through your school 🞏Known websites eg. e-Bug, BBC, Twinkl, abpi school

🞏Other 🞏I don’t access teaching resources

If other, please specify...................................................................................................................................................

**6a.** *(If ticked ‘social media groups‘)* **Which social media accounts do you currently use to find teaching resources or materials?**

🞏Twitter 🞏LinkedIn 🞏Pinterest 🞏Instagram 🞏Facebook 🞏Other

If other, please specify...................................................................................................................................................

**7.** *(unless ticked ‘I don’t access teaching resources‘ in Q.6)* **What teaching resources do you currently use?** e.g e-Bug or BBC bitesize

............................................................................................................................................................................

...........................................................................................................................................................................

**8.** *(unless ticked ‘I don’t access teaching resources‘ in Q.6)* **What device would you usually use to access resources?** (Tick all that apply)

🞏Laptop or PC 🞏Touch screen tablet 🞏ipad 🞏Mobile 🞏Other

If other, please specify...................................................................................................................................................

**9.** *(unless ticked ‘I don’t access teaching resources‘ in Q.6)* **How important is it that you are able to access educational resources on a range of devices?** (Tick **one**)

Unimportant Somewhat important Quite important Very Important Extremely important

🞏 🞏 🞏 🞏 🞏

## Ranking the importance of different website attributes

**In this section you will be asked to rank the importance of different attributes of websites. Please rank from most important to least important using numeric values starting with 1 (most important), please use each value only once. You will be asked to first rank a series of categories e.g appearance, and then sub-categories within this category, e.g images.**

Please note that you are not saying how good a particular site is with regard to each category, but rather how important each category is to you in deciding the overall usability of educational websites, from the perspective of an educator.

**10. Please RANK the importance of the categories (below) to you, as an educator accessing educational websites *(1 being the most important and 5 being the least important)*.**

| **Category** | **Rank**  **(1-5)** |
| --- | --- |
| **Appearance** (appealing and attractive to educators) |  |
| **Content** (educational website offers appropriate information for educators) |  |
| **Interactivity** (educational website provides the opportunity for its users to interact with the website producers and other users) |  |
| **Ease of use** (finding what you are looking for on a website is free of effort) |  |
| **Technical adequacy** (educational website functions as expected) |  |

**10i. Explain why you picked your chosen attribute as the most important (ranked at 1).**

**10ii. Explain why you picked your chosen attribute as the least important (ranked at 5)**

**10a. Please RANK the importance of the sub-categories of appearance (below) to you, as an educator accessing educational websites *(1 being the most important and 6 being the least important)*.**

| **Sub-category** | **Rank (1-6)** |
| --- | --- |
| **Colours** (attractive and appealing) |  |
| **Fonts** (appealing and legible) |  |
| **Media or graphics** (pictures and videos used appropriately and effectively to communicate the content) |  |
| **Page length** (avoids excessive scrolling) |  |
| **Style consistency** (consistent style and layout throughout the website) |  |
| **First impression** (the educational website has a good look and feel from initial viewing) |  |

**10b. Please RANK the importance of the sub-categories of content (below) to you, as an educator accessing educational websites *(1 being the most important and 6 being the least important)*.**

| **Sub-category** | **Rank (1-6)** |
| --- | --- |
| **Clarity** (content is understandable) |  |
| **Completeness** (educational website covers the depth and breadth of its subject area) |  |
| **Current and timely** (information is up to date) |  |
| **Relevance** (educational website offers content that is relevant to educators) |  |
| **Reliability and credibility** (educational website provides information that is trustworthy) |  |
| **Uniqueness** (educational website provides different information from its competitors) |  |

**10c. Please RANK the importance of the sub-categories of interactivity (below) to you, as an educator accessing educational websites *(1 being the most important and 2 being the least important)*.**

| **Sub-category**  **Explanation** | **Rank (1-2)** |
| --- | --- |
| **Sense of community** (educational website offers you the opportunity to be part of an online group or community eg. leave ratings/comments on resources for others to read, a forum, ‘email to a friend‘ option) |  |
| **Modern features** (educational website reflects the most current trend(s) eg. twitter feeds visible, blog posts) |  |

**10d. Please RANK the importance of the sub-categories of ease of use (below) to you, as an educator accessing educational websites *(1 being the most important and 5 being the least important)*.**

| **Sub-category** | **Rank (1-5)** |
| --- | --- |
| **Home-page indication** (educational website makes it obvious, clear and easy how to navigate back to the home-page) |  |
| **Navigation** (navigating the educational website is intuitive and easy to find the desired information) |  |
| **Learnability** (the educational website has a consistent navigation pattern which is easy to learn and remember) |  |
| **Guidance** (the educational website provides help for users in recovering from common errors or assist them in completion of tasks eg. FAQs, help option, search tool) |  |
| **Multi-language support** (educational website supports its user‘s language preferences) |  |

**10e. Please RANK the importance of the sub-categories of technical adequacy (below) to you, as an educator accessing educational websites *(1 being the most important and 4 being the least important)*.**

| **Sub-category** | **Rank (1-4)** |
| --- | --- |
| **Compatability with other devices** (educational website functions correctly on chosen device or browser) |  |
| **Load time** (pages or links load in an appropriate length of time) |  |
| **Valid links** (links function correctly and link to the expected pages) |  |
| **Limited use of special plug-ins** (educational website does not require the user to download special plug-ins eg. flashplayer) |  |

## The e-Bug Website

e-Bug is an on-going European project, operated by Public Health England, that creates health education resources for teachers and students, covering the subjects of: microbiology, hygiene and antibiotic resistance. We are looking to evaluate the teacher’s website to see what improvements are needed to make it the best website for our audience’s needs. We do hope you will therefore take just a few minutes to answer the following short questions.

**11. Have you used the e-Bug website before?** (Tick **one**)

🞏Yes 🞏No

**12.** *(If yes to 11.)* **How frequently do you visit e-Bug?** (Tick **one**)

Often Sometimes Every now and then Rarely

(At least once a week) (once a month) (a few times a year) (once a year or less)

🞏 🞏 🞏 🞏

**13.** *(If yes to 11.)* **Which of the following pages on the e-Bug website have you visited?** (Tick all that apply)

🞏Lesson plans 🞏Curriculum links 🞏Student pages 🞏Teacher news banner 🞏Teacher training module 🞏Project/partner information

**11a. How would you rate your computer skills?**

I am terrible I am bad I am ok I am a whizz

with computers with computers with computers with computers

🞏 🞏 🞏 🞏

## PART B: Familiarisation with e-Bug

**The following section will ask you to RATE e-Bug against some of the website attributes you have previously RANKED. It is therefore recommended that you browse e-Bug’s pages for teachers (not students) for approximately 5 minutes to (re)familiarise yourself with the website. It is advised that primary school educators focus on the junior pages and secondary school educators focus on the senior pages.**

**Please open the website in a separate window and after approximately five minutes, return to the window where select survey is open. Be aware that select survey may time out after 15 minutes if idle.**

**Copy this link:** [**http://www.e-bug.eu/**](http://www.e-bug.eu/)

**STOP HERE AND DO THE TASK COMPLETION WITH THE RESEARCHER!!**

## Rating e-Bug against different website attributes

**14. In regards to the e-Bug website, please indicate how strongly you agree with the following statements** (Tick **one** box for each statement).

|  | **Strongly disagree** | **Disagree** | **Neither agree or disagree** | **Agree** | **Strongly agree** |
| --- | --- | --- | --- | --- | --- |
| **Appearance** |  |  |  |  |  |
| The e-Bug website has **attractive and appealing colours**. | 🞏 | 🞏 | 🞏 | 🞏 | 🞏 |
| The e-Bug website uses **appealing and legible fonts**. | 🞏 | 🞏 | 🞏 | 🞏 | 🞏 |
| The e-Bug website **uses pictures and videos appropriately and effectively to communicate the content**. | 🞏 | 🞏 | 🞏 | 🞏 | 🞏 |
| The **pages** of the e-Bug website are of an **appropriate length** and **avoid excessive scrolling**. | 🞏 | 🞏 | 🞏 | 🞏 | 🞏 |
| The e-Bug website has a **consistent style and layout throughout its pages**. | 🞏 | 🞏 | 🞏 | 🞏 | 🞏 |
| The e-Bug website has a **good look and feel from first impression**. | 🞏 | 🞏 | 🞏 | 🞏 | 🞏 |
|  | **Strongly disagree** | **Disagree** | **Neither agree or disagree** | **Agree** | **Strongly agree** |
| **Content** |  |  |  |  |  |
| The content on e-Bug’s website is **understandable**. | 🞏 | 🞏 | 🞏 | 🞏 | 🞏 |
| The content on e-Bug’s website **covers the depth and breadth of microbiology, hygiene and antibiotic resistance**. | 🞏 | 🞏 | 🞏 | 🞏 | 🞏 |
| The content on e-Bug’s website is **current and timely**. | 🞏 | 🞏 | 🞏 | 🞏 | 🞏 |
| The content on e-Bug’s website is **relevant** to me as an educator. | 🞏 | 🞏 | 🞏 | 🞏 | 🞏 |
| The content on e-Bug’s website is **trustworthy**. | 🞏 | 🞏 | 🞏 | 🞏 | 🞏 |
| The content on e-Bug’s website is **different to its competitors**. | 🞏 | 🞏 | 🞏 | 🞏 | 🞏 |

| **Ease of use** |  |  |  |  |  |
| --- | --- | --- | --- | --- | --- |
| It is **obvious, clear and easy how to navigate back to e-Bug‘s home-page**. | 🞏 | 🞏 | 🞏 | 🞏 | 🞏 |
| **Navigating** the e-Bug website is **intuitive** and it is **easy to find your desired information**. | 🞏 | 🞏 | 🞏 | 🞏 | 🞏 |
| It is **easy to learn and remember the structure of e-Bug‘s website to locate specific pages**. | 🞏 | 🞏 | 🞏 | 🞏 | 🞏 |
| The e-Bug website **supports your language preference.** | 🞏 | 🞏 | 🞏 | 🞏 | 🞏 |
| **Technical adequacy** |  |  |  |  |  |
| The **pages and** **links** on the e-Bug **load in an appropriate length of time**. | 🞏 | 🞏 | 🞏 | 🞏 | 🞏 |
| The **links** on the e-Bug website **function correctly and link to the expected pages**. | 🞏 | 🞏 | 🞏 | 🞏 | 🞏 |

## Overall satisfaction

**16. How can the e-Bug website be improved?**

**17. Any other comments about the e-Bug website**

## Loyalty

**18. Would you use the e-Bug website in the future?** (Tick **one**)

🞏Yes 🞏No

If no, why not?

**19. Would you recommend the e-Bug website to friends or colleagues?** (Tick **one**)

🞏Yes 🞏No

If no, why not?

**20.** *(If yes to 18.)* **Which of the following pages are you likely to use in the future?** (Tick all that apply)

🞏Lesson plans 🞏Curriculum links 🞏Student pages 🞏Teacher news banner 🞏Teacher training modules 🞏Project/partner information

**21.** *(If ticked ‘lesson plans’ in 20)* **Which of the following topics are you likely to use?** (Tick all that apply)

**KS1 KS2 KS3 and 4**

🞏Introduction to microbes 🞏Introduction to microbes 🞏 Introduction to microbes

🞏Hand hygiene 🞏Useful Microbes 🞏Useful Microbes

🞏Respiratory hygiene 🞏Harmful Microbes 🞏Harmful Microbes

🞏Food hygiene 🞏Hand Hygiene 🞏Hand Hygiene

🞏Antibiotics 🞏Respiratory hygiene 🞏Respiratory hygiene

🞏Food hygiene 🞏Sexual Health

🞏Farm hygiene 🞏Immunity

🞏Immunity 🞏Vaccination

🞏Vaccination 🞏Antibiotic use and medicine

🞏Oral hygiene

🞏Antibiotic use and medicine  **KS5**

🞏Antibiotics

🞏Vaccination

**22.** *(If ticked ‘lesson plans’ in 20.)* **Which language(s) would you use the resources in?**

🞏English 🞏Other

If other, please specify...................................................................................................................................................

**25. Are there any topics relating to hygiene and health that you think we should include on the e-Bug website? Eg. history of medicine, vaccine development.**

## Additional content

**26. Please rate the importance of the following in relation to additional content that could be added to the e-Bug website** (Tick **one** box for each statement).

**How important is it for the e-Bug website to:**

|  | **Unimportant** | **Somewhat important** | **Quite important** | **Very important** | **Extremely important** |
| --- | --- | --- | --- | --- | --- |
| Have a search function on the menu bar e.g to search for a particular topic | 🞏 | 🞏 | 🞏 | 🞏 | 🞏 |
| Be able to leave feedback and see feedback from others on the e-Bug website? Eg. rate the lesson plans (similar to amazon and tripadvisor) | 🞏 | 🞏 | 🞏 | 🞏 | 🞏 |
|  |  |  |  |  |  |
| Be able to like, share on social media or email specific e-Bug resources to a friend | 🞏 | 🞏 | 🞏 | 🞏 | 🞏 |
| Be able to see the e-Bug twitter feed on the website homepage | 🞏 | 🞏 | 🞏 | 🞏 | 🞏 |
| Be able to view a blog and guest blog of invited e-Bug users on the e-Bug website | 🞏 | 🞏 | 🞏 | 🞏 | 🞏 |
| Be able to view FAQs or have a Q&A page on the e-Bug website | 🞏 | 🞏 | 🞏 | 🞏 | 🞏 |
| Have access to training opportunities on the e-Bug website? Eg. e-learning | 🞏 | 🞏 | 🞏 | 🞏 | 🞏 |
| Develop an app for users | 🞏 | 🞏 | 🞏 | 🞏 | 🞏 |

**27. Is there anything else you would like to see on the e-Bug website?**

## Thank you!

Thank you for taking the time to answer these questions. Your feedback will be vital to our evaluation of the website and, ultimately, making e-Bug better for you.

If you would like to stay up-to-date on all things e-Bug, sign up for the termly newsletter.

**30.** 🞏Sign up for termly e-Bug newsletter 🞏Not interested

*If yes to newsletter:*

**Name.............................................................................................................................................................................**

**Email address...............................................................................................................................................................**

**Job role.........................................................................................................................................................................**

**School Name............................................................................................................................................................**

**31. Would you be interested in receiving a £5 gift voucher and your name entered into a draw to win a pack of 5 giant microbes?** (Tick **one** from each statement)

i. 🞏Yes please to £5 gift voucher OR 🞏Please donate my £5 to further research

ii. 🞏Yes, please enter my name into the OR 🞏No thank you to giant microbes

draw to win a pack of 5 giant microbes

*If yes to Q31 i or ii:*

***31a. Please provide your full name and address of where you would like the gift voucher and/or giant microbes to be sent***

***....................................................................................................................................................................................................................................................................................................................................................................................................................................................................................................................................................................................***

***
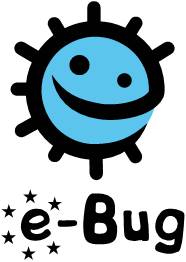
***

Thank you for taking the time to answer these questions!
