# Supplementary material for: Evaluation of an Educational Health Website on Infections and Antibiotics in England: Mixed Methods, User-Centered Approach
Source: JMIR Form Res. 2020 Apr 6;4(4):e14504. doi: 10.2196/14504 (PMC7171564; doi:10.2196/14504)
Supplement: Multimedia Appendix 2 [file formative_v4i4e14504_app2.docx]

| Junior educator: Task 1  🡪You have misplaced your hard copy of the full pack of Key stage 2 e-Bug lesson plans and resources, but were told that you could download all of the resources in one go from the e-Bug website. Locate and click on the link to the PDF version of this. |
| --- |
| Junior educator: Task 2  🡪In your junior school, you would like to teach a lesson on how microbes can spread from person to person simply by shaking each other’s hands; which will lead on to how best to wash your hands. You live in England. Locate and click on the link to the ‘complete pack’- word version of this. |
| Junior educator: Task 3  🡪You are teaching a lesson on vaccinations and would like to see which vaccines your pupils are recommended to have had by the time they’re in primary school. Locate and click on the relevant link. |
| Junior educator: Task 4  🡪You want to teach a lesson on antibiotic resistance and remember seeing a colleague with a worksheet for the pupils where they have to match the words to their correct meaning. Locate and open this resource. |
| Junior educator: Task 5  🡪 You think that e-Bug has lots of great resources, but want to see an overview of how they fit into the national curriculum for KS2 in England. Locate and click on the relevant link. |
| Junior educator: Task 6  🡪Locate back to the e-Bug home-page which looks like this:  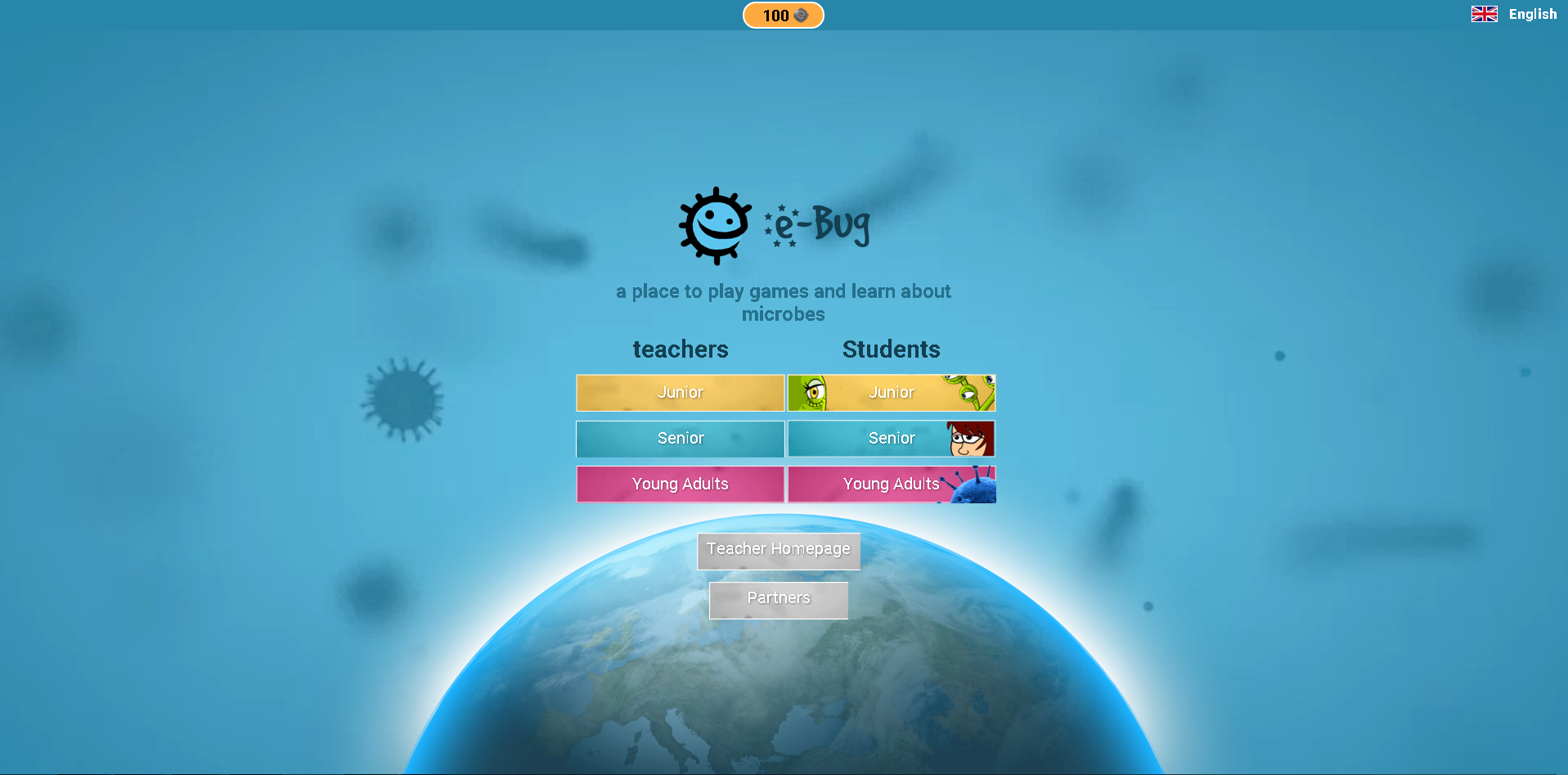 |
| Junior educator: Task 7  🡪 You want to save on printing, so didn’t print out the full pack of resources for Key stage 2 that you located in Task 1. But can you find it again? |

| Senior educator: Task 1  🡪You have misplaced your hard copy of the full pack of senior school e-Bug lesson plans and resources, but were told that you could download all of the resources in one go from the e-Bug website. Locate and click on the link to the PDF version of this. |
| --- |
| Senior educator: Task 2  🡪In your senior school, you would like to teach a lesson on how poor hand hygiene can lead to the spread of microbes and disease. Locate and click on the link to the ‘complete pack’- word version of this. |
| Senior educator: Task 3  🡪You are teaching a lesson on vaccinations and would like to see which vaccines your pupils are recommended to have had by the time they’re in secondary school. Locate and click on the relevant link. |
| Senior educator: Task 4  🡪In your senior school, you want to teach a lesson on antibiotic resistance and remember seeing a colleague with a worksheet for the pupils about mixing up test results whilst working at a local laboratory on work experience. Locate and open this resource. |
| Senior educator: Task 5  🡪 You think that e-Bug has lots of great resources, but want to see an overview of how they fit into the national curriculum for KS3 in England. Locate and click on the relevant link. |
| Senior educator: Task 6  🡪Locate back to the e-Bug home-page which looks like this:  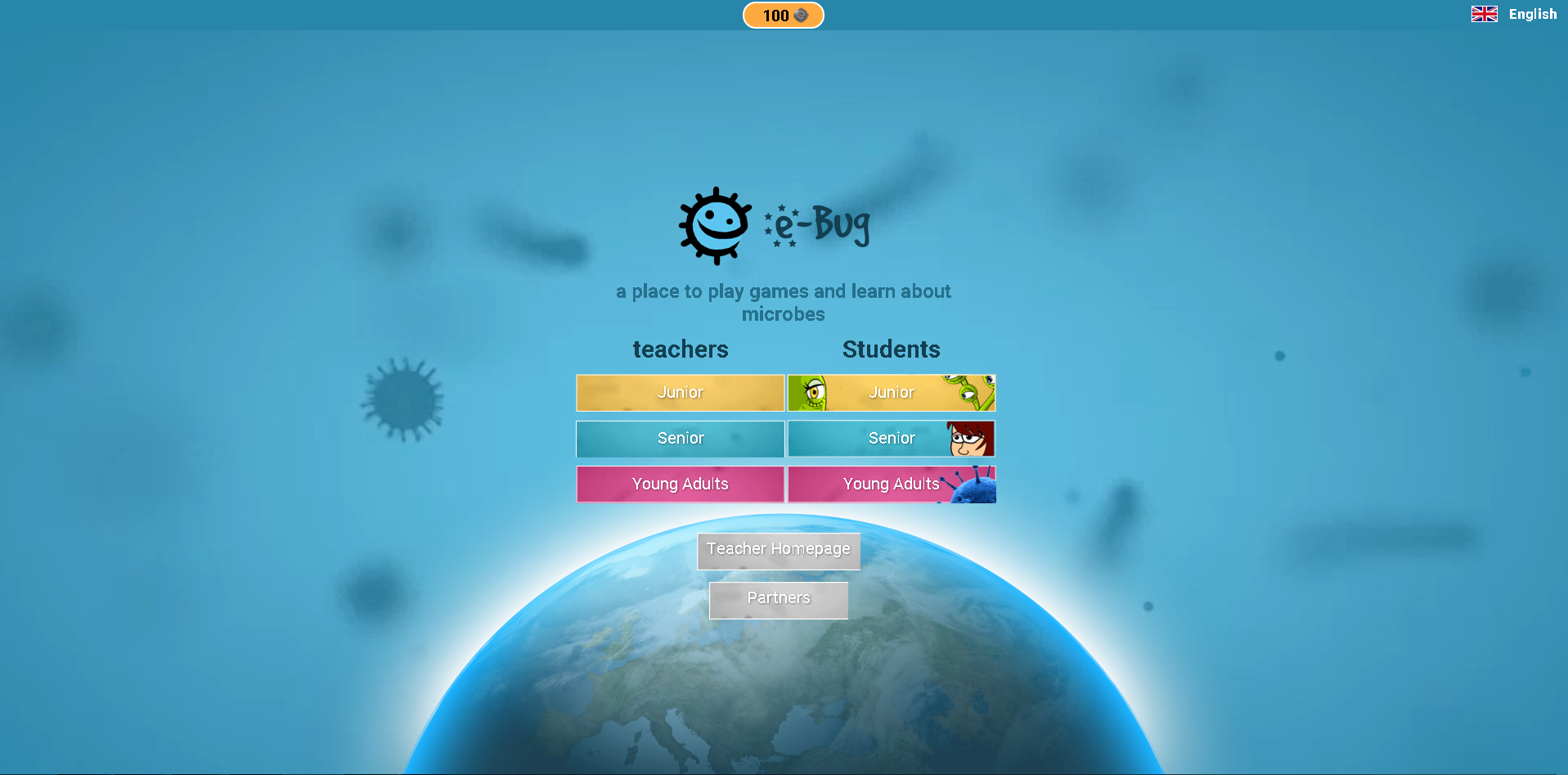 |
| Senior educator: Task 7  🡪 You want to save on printing, so didn’t print out the full pack of resources for Key stage 3 that you located in Task 1. But can you find it again? |
