# Supplementary material for: Evaluation of an Educational Health Website on Infections and Antibiotics in England: Mixed Methods, User-Centered Approach
Source: JMIR Form Res. 2020 Apr 6;4(4):e14504. doi: 10.2196/14504 (PMC7171564; doi:10.2196/14504)
Supplement: Multimedia Appendix 3 [file formative_v4i4e14504_app3.docx]

| **Role at the school**  **(n=106)** | | **Age range that they teach** | | **Gender** | | **Age Range of educator (years)** | | **Previous use of e-Bug** | | **Self-measure of computer skills** | |
| --- | --- | --- | --- | --- | --- | --- | --- | --- | --- | --- | --- |
| Teacher | 86 | KS1 | 21 | Female | 84 | 24 or under | 11 | Yes | 36 | I am terrible with computers | 0 |
| Teaching assistant | 6 | KS2 | 37 | Male | 20 | 25-35 | 27 | No | 70 | I am not very good with computers | 6 |
| Science technician | 4 | KS3 | 68 | Would rather not say | 1 | 36-45 | 29 |  |  | I am ok with computers | 72 |
| Head Teacher | 2 | KS4 | 63 | Blank | 1 | 46-55 | 27 |  |  | I am a computer whizz | 27 |
| Assistant head | 1 | KS5 | 52 |  |  | 56 or over | 12 |  |  | Blank | 1 |
| Trainee teacher | 2 |  |  |  |  |  |  |  |  |  |  |
| Consultant | 1 |  |  |  |  |  |  |  |  |  |  |
| Freelance teacher trainer | 1 |  |  |  |  |  |  |  |  |  |  |
| Lecturer | 1 |  |  |  |  |  |  |  |  |  |  |
| School Nurse | 1 |  |  |  |  |  |  |  |  |  |  |
| Blank | 1 |  |  |  |  |  |  |  |  |  |  |

| **Frequency of e-Bug use (n=36)** | |
| --- | --- |
| Often (at least once a week) | 2 |
| Sometimes (once a month) | 9 |
| Every now and then (a few times a year) | 18 |
| Rarely (once a year or less) | 5 |
| Blank | 2 |

| **Device used to access Resources** | |
| --- | --- |
| Laptop or PC | 101 |
| ipad | 35 |
| Mobile | 17 |
| Touch screen tablet | 14 |
| Interactive whiteboard | 2 |
| Hue visualizer | 1 |
| Magazines, Books | 1 |
| Chrome book | 1 |
| Blank | 3 |
