# Supplementary material for: Evaluation of an Educational Health Website on Infections and Antibiotics in England: Mixed Methods, User-Centered Approach
Source: JMIR Form Res. 2020 Apr 6;4(4):e14504. doi: 10.2196/14504 (PMC7171564; doi:10.2196/14504)
Supplement: Multimedia Appendix 4 [file formative_v4i4e14504_app4.docx]

“Having taught from the books, I would find what I need then locate it on the website.”

“Like timeline because personal to them, relevant, and I haven’t come across anything like that.”

“Useful and fun link [vaccination timeline]. Applicable to students and more relatable, so may be more useful to them. They would be interested in EU countries to compare with people from other countries. And interested to see what they should get if travelling to other countries.”

“I think it is great and helpful for teaching the topics. It is quick to glean the information needed and to know where to go. The vaccination timeline and comparison links are good too. I like that the resources can all be accessed singly as well as downloaded in packs.”

“Stuff on there is really student friendly and teacher friendly etc. Good that in word and PDF. Word to edit for students.”

“A fantastic resource for anyone wishing to teach about microbes, comprehensively.”

“Great website which has been highly effective in supporting the new science GCSE.”

“Definitely a niche in the market for it. We have history of medicine and urology but useful to have a site that does all and that makes it applicable and user friendly for children.”

“Animations are hard to get. BBC bitesize are not great. Want it to match with resources. Play clip and then use the resources that fit in… Youtube is either incredibly simplified or too far than they have to know. Good to have something that is consistent and uses same kind of language and link to worksheets. Animations look good.”

“Looks like a great resource, I shall look forward to embedding some of these resources into my lessons. Thanks.”

“Links to curriculum are really good.”

“Debate kits good. Prepare them well for question to complete in exam (6 mark). Good to engage and discuss.”

“Very easy to use, especially if not computer literate. Easy to read font. On the whole, relatively straight forward. Not too much scrolling which personally prefer. Would rather more clicks and less information on each page, which e-Bug has.”

“Best I've seen :-)”
