# Supplementary material for: Evaluation of an Educational Health Website on Infections and Antibiotics in England: Mixed Methods, User-Centered Approach
Source: JMIR Form Res. 2020 Apr 6;4(4):e14504. doi: 10.2196/14504 (PMC7171564; doi:10.2196/14504)
Supplement: Multimedia Appendix 5 [file formative_v4i4e14504_app5.docx]

| **1^st^**  **Content** | **Reliability and credibility** (educational website provides information that is trustworthy) | **1^st^** |
| --- | --- | --- |
|  | **Clarity** (content is understandable) | **2^nd^** |
|  | **Relevance** (educational website offers content that is relevant to educators) | **3^rd^** |
|  | **Completeness** (educational website covers the depth and breadth of its subject area) | **4^th^** |
|  | **Current and timely information** (information is up to date) | **5^th^** |
|  | **Uniqueness** (website provides different information from its competitors) | **6^th^** |
| **2^nd^**  **Ease of Use** | **Navigation** (navigating the educational website is intuitive and easy to find the desired information) | **1^st^** |
|  | **Learnability** (the educational website has a consistent navigation pattern which is easy to learn and remember) | **2^nd^** |
|  | **Home-page indication**(educational website makes it obvious, clear and easy how to navigate back to the home-page) | **3^rd^** |
|  | **Guidance**(the educational website provides help for users in recovering from common errors or assist them in completion of tasks eg. FAQs, help option, search tool) | **4^th^** |
|  | **Multi-language support**(educational website supports its user‘s language preferences) | **5^th^** |
| **3rd**  **Interactivity** | **Modern features** (educational website reflects the most current trend(s) eg. twitter feeds visible, blog posts) | **1^st^** |
|  | **Sense of community** (educational website offers you the opportunity to be part of an online group or community eg. leave ratings/comments on resources for others to read, a forum, ‘email to a friend‘ option) | **2^nd^** |
|  | **Load time** (pages or links load in an appropriate length of time) | **1^st^** |
| **4^th^**  **Technical adequacy** | **Valid links** (links function correctly and link to the expected pages) | **2^nd^** |
|  | **Compatibility with other devices** (educational website functions correctly on chosen device) | **3^rd^** |
|  | **Limited use of special plug-ins** (website does not require the user to download special plug-ins eg. flashplayer) | **4^th^** |
| **5th**  **Appearance** | **Media or graphics** (pictures and videos used appropriately and effectively to communicate the content) | **1^st^** |
|  | **First impression** (educational website has a good look and feel from initial viewing) | **2^nd^** |
|  | **Fonts** (appealing and legible) | **3^rd^** |
|  | **Page length** (avoids excessive scrolling) | **4^th^** |
|  | **Style consistency** (consistent style and layout throughout the website) | **5^th^** |
|  | **Colours** (attractive and appealing) | **6^th^** |
