# Supplementary material for: Evaluation of an Educational Health Website on Infections and Antibiotics in England: Mixed Methods, User-Centered Approach
Source: JMIR Form Res. 2020 Apr 6;4(4):e14504. doi: 10.2196/14504 (PMC7171564; doi:10.2196/14504)
Supplement: Multimedia Appendix 6 [file formative_v4i4e14504_app6.docx]

| **Navigation** (navigating the educational website is intuitive and easy to find the desired information)  “Would be useful, that when on page, the link in the menu goes a different colour. Or when on a certain page, deactivate the link in the menu (so you know you’ve actually gone to that page).”  “Want the left hand menu to break up and say resources etc (similar to top menu).”  “The front page belies the complexity of the content. A tree index would be nice to have an overview of what lies hidden. Obviously, with practice you get to know a site well, but a newcomer would be unaware of the volume and scope of material.” |
| --- |
| **Reliability and credibility** (educational website provides information that is trustworthy)  “Cite where the information given has come from. Clearly show the year the information was updated.” |
| **Relevance** (educational website offers content that is relevant to educators)  “It would be useful to have more KS4 resources and links to the KS4 specifications.” |
| **Completeness** (educational website covers the depth and breadth of its subject area)  “More resources.” |
| **Sense of community** (educational website offers you the opportunity to be part of an online group or community eg. leave ratings/comments on resources for others to read, a forum, ‘email to a friend‘ option)  “An area where users can chat and rate resources.”  “A link to healthcare professionals and discussion pages with research articles.”  “Finding partners to discuss regional and national differences.” |
| **Home-page indication** (educational website makes it obvious, clear and easy how to navigate back to the home-page)  “Would never think to click on the logo. Have a pop-up description when hover over with mouse. Call teacher’s home page, teacher’s hub.”  “Have the word home in the logo. Call teacher’s section- teacher’s page.”  “Everything else is words so wasn’t looking for a symbol.”  “Not consistent with ‘young adult page’. When scrolling over, it doesn’t come up with ‘hand image’- noticed when surfing the websites. And this does not take you back to the e-Bug home page, so very confusing. If the e-Bug logo moved, or when hovered over it, it got bigger or said “return home”.”  “Expected home link to take to the e-Bug home. Suggests another tab at the top saying ‘e-Bug home page’.” |
| **Compatibility with other devices** (educational website functions correctly on chosen device)  “More accessible from mobile devices.”  “An e-bug app for use on i-pads would be amazing as I adore teaching with your resources.”  “Presumed it wasn’t the final production of the website. On the homepage you have to scroll before you even see anything [as website not responsive]. Should be able to see all on screen.” |
| **Valid links** (links function correctly and link to the expected pages)  “Would be useful for full-pack to have working links and content page linked to sections.”  “Perhaps when hover mouse over, dialogue box with info on each link could come up.”  “Perhaps when hover mouse over link, a dialogue box with info on each link could come up.”  “Would be useful to have a print-screen or image of what the worksheet looked like. Or rollover image… interactive feature.”  “Was expecting a heading that said “lesson plans and resources”. Didn’t know that “pack” meant lesson plans and resources.” |
| **Media or graphics** (pictures and videos used appropriately and effectively to communicate the content)  “Videos need to work.”  “The graphics are a bit dated. I think children expect a higher standard as they are used to in video games.” |
| **Fonts** (appealing and legible)  “Titles need to be a bigger font size so they stand out more.”  “The font: utilising the space more effectively…lots of empty space on the screen at the sides.” |
| **Colours** (attractive and appealing)  “Not using yellow as it is hard to see.”  “Change the background colour from white to a pale colour as dyslexic children can find this difficult to view. Please don’t switch it to yellow on blue or visa versa.”  “Blue writing on blue is very difficult to read on homepage.”  “Blue and orange are contrasting colour schemes, but may need to test with dyslexic people. Blue and red are difficult for them to see.” |
| **Page length** (avoids excessive scrolling)  “Participant did not think to scroll. Need to be able to see that there is more information without scrolling.” |
| **Style consistency** (consistent style and layout throughout the website)  “Noticed that some resources opened a new tab and some linked to a new page. Would be better for all to do the same (not new tab).”  “Wanted consistency of left hand menu, even on teacher’s home-page.” |
| **Multi-language support** (educational website supports its user‘s language preferences)  “Explicit links to skills in Welsh national curriculum.”  “Add information relevant to the Northern Ireland curriculum in particular, (as for England and Scotland).” |
| **Interactivity** (educational website provides the opportunity for its users to interact with the website producers and other users)  “Could there something like a log or protocol to show which parts I've done successfully? That might be motivational in the process of self-responsible learning.”  “Usernames for pupils and teachers.”  “More interactive lessons.” |
| **Appearance** (appealing and attractive to educators)  “Buttons look pasted on: remove borders. Don’t need horizontal scroller (when viewing on smaller laptop screen). Not sure how necessary button to get back to top of website is as not large enough website for it to be needed.” |
| Other ideas  “Sound.”  “Graded mark schemes and more detailed assessments.”  “Links with exam questions on relevant topics could be included.”  “Resources and activities in conjunction/partnership with known health organisations e.g. NHS.”  “Maybe some CPD days and events for pupils.”  “Guest speakers booking section.”  “Would like a ‘print this page’ option on each page of full pack.”  “The wording KS3 threw me. Assumed senior was all senior key stages. Or have 3 links for the different key stages.”  “Label it as ‘KS3 lesson pack’. Label whether KS3 or KS4 throughout website- in hyperlinks.”  “[on e-Bug home page] When it says teacher, thought it meant my age as a teacher, so would have clicked young adult. Would be more useful to work in Key Stages ie. KS3.”  “What is the difference between young adult (KS5 or 6th form) and senior. To me, young adult means university.” |
